# Regional Myocardial Work Measured by Echocardiography for the Detection of Myocardial Ischemic Segments: A Comparative Study With Invasive Fractional Flow Reserve

**DOI:** 10.3389/fcvm.2022.813710

**Published:** 2022-03-16

**Authors:** Ying Guo, Chenguang Yang, Xiang Wang, Zuowei Pei, Huolan Zhu, Xuyang Meng, Ziyu Zhou, Xiaotong Lang, Sun Ning, Ruisheng Zhang, Fang Wang

**Affiliations:** ^1^Department of Cardiology, Beijing Hospital, National Center of Gerontology, Beijing, China; ^2^Institute of Geriatric Medicine, Chinese Academy of Medical Sciences, Beijing, China; ^3^Graduate School of Peking Union Medical College, Chinese Academy of Medical Sciences, Beijing, China; ^4^Department of Gerontology, Shanxi Provincial People's Hospital, Shanxi Provincial Clinical Research Center for Geriatric Medicine, Xi'an, China

**Keywords:** myocardial work, fractional flow reserve, regional myocardial work, coronary artery disease, single-vessel stenosis

## Abstract

**Purpose:**

This study is to assess the diagnostic value of noninvasive regional myocardial work (MW) by echocardiography for detecting the functional status of coronary stenosis using fractional flow reserve (FFR) as a standard criterion.

**Methods:**

A total of 84 consecutive patients were included in this study, among which 92 vessels were identified with ≥50% stenosis confirmed by invasive coronary angiography. Patients were investigated by invasive FFR and transthoracic echocardiography. Regional MW indices including myocardial work index (MWI), myocardial constructive work (MCW), myocardial wasted work, and myocardial work efficiency were calculated.

**Results:**

MWI and MCW were significantly impaired in the FFR ≤ 0.75 group compared with the FFR > 0.75 group (both *p* < 0.01). There were significant positive associations between MWI and MCW with FFR. In total group, MWI <1,623.7 mmHg% [sensitivity, 78.4%; specificity, 72.2%; area under the curve value, 0.768 (0.653–0.883)] and MCW <1,962.4 mmHg% [77.0%; 72.2%; 0.767 (0.661–0.872)], and in single-vessel subgroup, MWI <1,412.1 mmHg% [93.5%; 63.6%; 0.808 (0.652–0.965)] and MCW <1,943.3 mmHg% [(84.8%; 72.7%; 0.800 (0.657–0.943)] were optimal to detect left ventricular segments with an FFR ≤ 0.75. MWI and MCW significantly increased after percutaneous coronary intervention in 13 cases.

**Conclusion:**

In patients with coronary artery disease, especially those with single-vessel stenosis, the regional MW measured by echocardiography exhibited a good diagnostic value in detecting significant myocardial ischemia compared to the standard FFR approach.

## Introduction

For patients with coronary artery disease (CAD), assessment of the functional significance of intermediate coronary atherosclerotic plaques remains challenging. Fractional flow reserve (FFR) is the gold standard for the evaluation of coronary lesion-related ischemia. However, FFR may not be a method of preference for patients who do not need coronary intervention. Therefore, non-invasive imaging techniques for functional assessment of coronary arterial stenosis have been developed (1). Although CT and cardiac MRI are promising non-invasive methods for the assessment of CAD, echocardiography is still a first-line diagnostic tool because of its feasibility and reliability. In recent years the diagnostic power of echocardiography has evolved with the development of new techniques (2). Regional myocardial dysfunction caused by coronary stenosis can be detected by two-dimensional speckle tracking echocardiography (3, 4). In stable patients, the longitudinal strain is useful for the detection of functionally significant CADs, which has been confirmed by invasive FFR (5–7). However, because of the load-dependent nature of myocardial strain, the interpretation of the myocardial functional status based on strain analysis has to be made in a context of ventricular loading conditions (8). Non-invasive myocardial work (MW) is a novel technique for assessing cardiac function. MW accounts for deformation and afterload and provides incremental value to the evaluation of cardiac function (9–11). MW also correlates favorably with invasive coronary angiography for measures of coronary stenosis severity (12). However, the agreement between MW and a gold standard diagnostic tool in the functional assessment of myocardial segments supplied by stenotic coronary arteries has yet to be reported. The objective of the current study is to investigate the association between MW and FFR in a cohort of clinically suspected CAD patients.

## Materials and Methods

### Patients

This study retrospectively analyzed the data of 84 consecutive patients with clinically suspected CAD who visited Beijing Hospital (Beijing, China) between November 2018 and December 2021. From the coronary angiography, we identified 92 vessels with lumen stenosis. Intermediate stenosis was defined as 40–75% lumen stenosis (13, 14). FFR for each stenotic vessel was measured. The inclusion criteria were as follows: (1) patients with myocardial ischemia-related symptoms or positive examination results; (2) age >18 years; (3) sinus rhythm. The exclusion criteria were as follows: (1) left ventricular ejection fraction (LVEF) <55%; (2) abnormal motion of regional wall at rest; (3) left main CAD involved; (4) previous myocardial infarction with total occlusion or severe stenosis and confirmed collateral flow; (5) any pathologies that result in an obstruction or pressure gradient between the aorta and left ventricular (LV); (6) severe valvular heart disease or arrhythmia; (7) poor image quality for speckle tracking.

### Invasive Coronary Angiography and FFR Measurements

All patients underwent an invasive coronary angiography with FFR. Coronary pressure measurements were routinely collected during heart catheterization to assess the functional severity of the intermediate coronary stenosis. During the procedure, a 6F angiography catheter was inserted through the radial artery or femoral artery for selective left and right coronary angiography. The baseline coronary angiogram was acquired in multiple projections.

After the angiography, a guiding catheter of more than 6F was inserted to the target vessel. A pressure–temperature sensor-tipped 0.014-inch guidewire (St. Jude Medical, USA), previously flushed and calibrated to zero pressure, was advanced to the tip of the guiding catheter where an equalization was performed to ensure identical pressures between the guiding catheter and the pressure guidewire (15). The FFR pressure guidewire was then placed at least 3–4 cm below the target stenosis. The FFR guidewire was manipulated until an optimal and stable velocity signal was obtained (16). Mean aortic blood pressure (Pa) and mean intracoronary blood pressure distal to the target stenosis (Pd) were simultaneously measured through the guiding catheter and the pressure guidewire, at both baseline and during sustained hyperemia, which was achieved by intravenous infusion of adenosine at rate of 0.14–0.18 mg·kg-1/min for at least 60 s. FFR was interpreted jointly by an interventional cardiologist and a technician who were both blinded to the echocardiographic result. FFR was calculated by dividing mean post-stenotic coronary pressure by mean aortic blood pressure (Pd/Pa). Two FFR cutoff values, ≤ 0.75 (17, 18) and ≤ 0.80 (6, 19), were considered functionally significant. Quantitative coronary angiography (QCA) was performed by independent technicians who were instructed to do FFR measurement at specific location but were blinded to the result of FFR and the other information. The percentage of diameter stenosis was analyzed using a QCA software (Centricity Cardiology CA1000, GE Healthcare) (20).

### Echocardiography

Baseline echocardiography was performed before the coronary angiography when patients were admitted to hospital. The echocardiographic studies were conducted by experienced sonographers using a Vivid E95 ultrasound system equipped with a M5S transducer (GE Vingmed Ultrasound, Horten, Norway). Images were acquired and stored in cine loop format for offline analysis using the EchoPac software (EchoPac 203, GE Vingmed Ultrasound). All measurements were performed according to the American Society of Echocardiography guidelines (21, 22). LVEF was calculated using the biplane Simpson's method. Global longitudinal strain (GLS) of the LV was the average peak systolic longitudinal strain of three apical views (23).

### MW Parameters

In the Echopac software, MW indices were obtained using a pressure-strain loop (PSL) area module, which was constructed from non-invasively estimated LV pressure curves and LV strain. Peak systolic LV pressure was assumed to be equal to the peak brachial cuff systolic blood pressure that was measured simultaneously at the echocardiography examination. This method has been validated in many studies (8, 24–27). The myocardial work index (MWI) represents the total work within the area of the PSL during the time interval from mitral valve closure to mitral valve opening. Additional parameters were calculated as follows: myocardial constructive work (MCW), the myocardial work performed for shortening during ventricular systole and for lengthening during isovolumic relaxation; myocardial wasted work (MWW), the myocardial work performed for lengthening during ventricular systole and for shortening during isovolumic relaxation; myocardial work efficiency (MWE), the percentage of myocardial constructive work in total myocardial work [MCW/(MCW + MWW)]. Under the MW algorithm in the EchoPac software, the LV was divided into 18 segments (6 x basal, 6 x midventricular, and 6 x apical segments). Standardized myocardial segmentation was adopted (21, 28, 29). The values of the regional MWI, MWE, MCW, MWW for each stenotic coronary artery were calculated as the average of the corresponding segments belonging to each region (29). Some echocardiography and MW assessments were repeated for patients who underwent percutaneous coronary intervention (PCI).

### Statistical Analysis

Continuous variables were expressed as mean ± SD and categorical variables as numbers and percentages. The normality of the distribution was tested using the Kolmogorov–Smirnov test. The comparison of normally distributed variables between two groups was performed using an independent-sample *t*-test. Among LV segments perfused by vessels with an FFR ≤ 0.75, 0.76–0.80, and >0.80, MW parameters were compared using One-way ANOVA followed by Tukey's HSD post-hoc test. Comparison of non-normally distributed variables was performed using a Mann-Whitney *U* test. A paired *t*-test was used to compare the MW parameters before and after PCI. A χ^2^ or Fisher exact-test was used for categorical data. Pearson's correlation was used to test the correlation between FFR and MW parameters. To test the diagnostic accuracy and determine the cut-off values of MW in detecting LV segments perfused by vessels with FFR ≤ 0.75 and FFR ≤ 0.80, receiver operator characteristics (ROC) analysis was performed. Intra and inter-observer variabilities were calculated using intraclass correlation coefficients (ICCs). A *p* < 0.05 was considered statistically significant. Statistical analyses were performed using the SPSS version 23.0 software (IBM Corp, Armonk, NY).

## Results

### Baseline Characteristics

**Figure 1:**
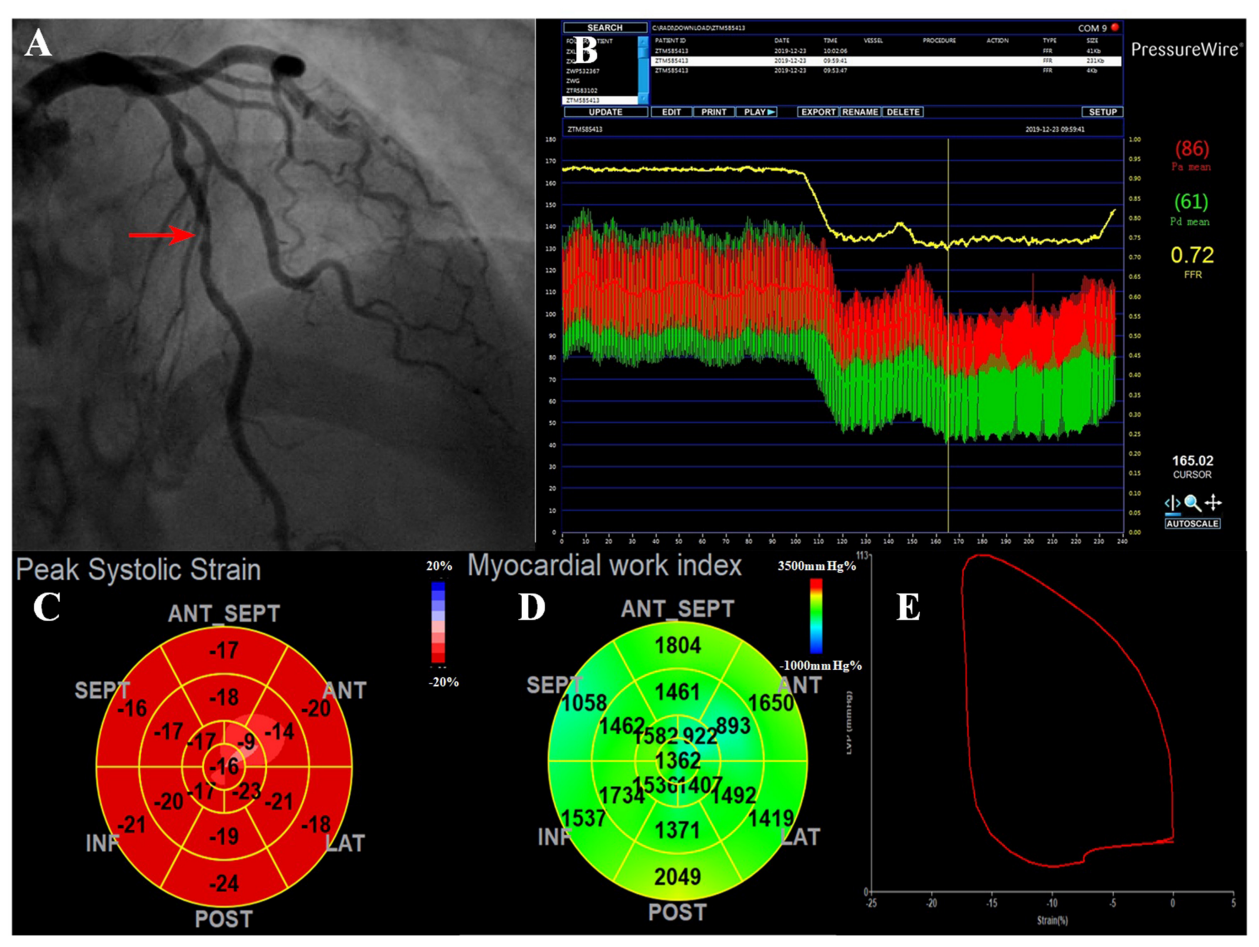
A representative case of the ischemic LAD with intermediate stenosis. **(A)** Invasive coronary angiogram demonstrates 50% narrowing in the LAD (red arrow). **(B)** The corresponding FFR value was 0.72, which was below the ischemic threshold of 0.75, indicating a functionally significant stenosis. **(C)** The bull's eye plot shows decreased peak systolic longitudinal strains in the apex-anterior, mid-anterior segments. **(D)** MWI values were impaired mostly in the anterior wall (supplied by the LAD). The regional MWI was 1,411.62 mmHg% and MCW was 1,686.62 mmHg%. **(E)** LV pressure-strain loop diagram. FFR, fractional flow reserve; LAD, left anterior descending; MCW, myocardial constructive work; MWI, myocardial work index.

The baseline clinical, angiographic, and echocardiographic characteristics are displayed in Table 1. In total, 84 patients (92 vessels) were included in this study. Fifty-seven patients had only one stenotic coronary artery branch and 27 patients had multiple-vessel stenosis (≥ 2 diseased coronary branches). A total of 118 vessels were involved in 84 patients, of which 92 vessels were examined for FFR. The mean invasive FFR value was 0.82 ± 0.08, and the mean percentage of stenosis was 52.7 ± 9.1%. FFR values of ≤ 0.75 were found in 18/92 (19.6%) of total vessels and ≤ 0.8 in 32/92 (34.8%) of total vessels. Diabetes mellitus was diagnosed in 7 patients with an FFR ≤ 0.75 and in 13 patients with an FFR > 0.75 (63.6 vs. 28.3%, *p* = 0.034). Between the two FFR subgroups, there were no significant differences regarding systolic blood pressure (BP) (total vessel: 127.3 ± 15.9 vs. 134.3 ± 16.0 mmHg, *p* = 0.096; single vessel: 122.2 ± 11.5 vs. 131.3 ± 16.6 mmHg, *p* = 0.092), diastolic BP (total vessel: 75.3 ± 9.8 vs. 76.9 ± 11.0 mmHg, *p* = 0.557; single vessel: 77.5 ± 10.7 vs. 75.9 ± 10.6 mmHg, *p* = 0.667), and heart rate (total vessel: 79.0 ± 13.7 vs. 77.6 ± 12.3 bpm, *p* = 0.678; single vessel: 76.2 ± 13.2 vs. 77.1 ± 13.8 bpm, *p* = 0.848). No significant differences were observed in other clinical (age, sex ratio, hypertension, hyperlipidemia, smoking, family history of CAD) or echocardiographic characteristics (LV end-diastolic dimension, LV mass index, LV wall thickness, right atrial volume, E wave, A wave, E/A ratio, E/e' ratio and LVEF) between the two FFR subgroups (FFR ≤ 0.75 vs. > 0.75), in both total-vessel and single-vessel groups. 67.9% of the patients had a single vessel involved, 23.8% had two vessels involved, and the rest (8.3%) had three vessels involved. The left anterior descending (LAD) vessel was the most frequently involved vessel. A typical MW pattern of a patient with LAD stenosis is shown in Figure 1.

**Table 1:** General characteristics and angiographic and echocardiographic data in the whole cohort of patients.

**Characteristics**	**Total**
	**(*n* = 84)**
Age, years	64.5 ± 10.3
Male, *n* (%)	52 (61.9)
Hypertension, *n* (%)	45 (53.6)
Hyperlipidemia, *n* (%)	54 (64.3)
DM, *n* (%)	33 (39.3)
Smoking, *n* (%)	46 (54.8)
Family history of CAD, *n* (%)	34 (40.5)
BMI, kg/m^2^	25.9 ± 3.4
HR, bpm	77.8 ± 12.9
Systolic BP, mmHg	132.4 ± 16.2
Diastolic BP, mmHg	76.6 ± 10.6
**Symptoms**	
Asymptomatic/silent ischemia, *n* (%)	15 (17.9)
Stable angina, *n* (%)	42 (50.0)
Acute coronary syndromes, *n* (%)	27 (32.1)
**Angiographic characteristics**	
Diameter stenosis, %	52.7 ± 9.1
LAD stenosis, *n* (%)	79 (94.0)
LCX stenosis, *n* (%)	19 (22.6)
RCA stenosis, *n* (%)	20 (23.8)
**Echocardiographic characteristics**	
LVEDD, mm	45.7 ± 3.1
Septal wall thickness, cm	1.0 ± 0.1
Posterior wall thickness, cm	1.0 ± 0.1
LV mass/BSA, g/m^2^	88.8 ± 19.6
Right atrial volume, ml	25.3 ± 5.1
E wave, m/s	0.7 ± 0.2
A wave, m/s	0.9 ± 0.2
E/A ratio	0.9 ± 0.3
E/e' ratio	12.0 ± 3.6
LVEF, %	63.9 ± 3.5

*Data are expressed as mean ± SD when appropriate. BP, blood pressure; BSA, body surface area; CAD, coronary artery disease; DM, diabetes mellitus; LAD, left anterior descending; LCX, left circumflex; LV, left ventricular; LVEDD, left ventricular end-diastolic dimension; LVEF, left ventricular ejection fraction; RCA, right coronary artery*.

The single-vessel stenosis subgroup was comprised of 57 subjects. 91.2, 5.3, and 3.5% of the patients had LAD stenosis, left circumflex (LCX) stenosis, and right coronary artery (RCA) stenosis, respectively. The mean invasive FFR value was 0.82 ± 0.08, and the mean percentage of stenosis was 53.0 ± 9.2%. In the single-vessel stenosis subgroup, the percentage of stenosis in patients with FFR ≤ 0.75 was increased compared to those in patients with FFR > 0.75 (63.2 ± 13.4% vs. 50.5 ± 5.9%, *p* = 0.011).

### Analysis of MW in Segments With and Without Reduced FFR

**Figure 2:**
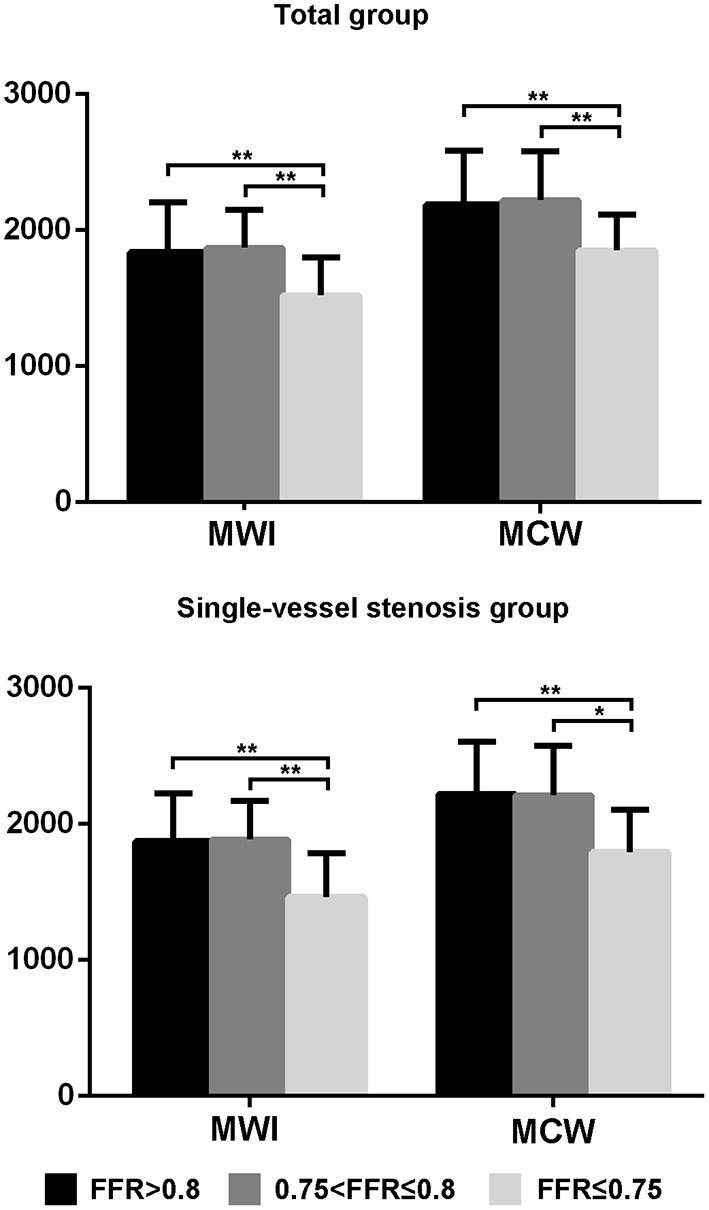
The MWI and MCW values of left ventricular segments with FFR ≤ 0.75, 0.76–0.80, and >0.80 in the total-vessel group and single-vessel stenosis subgroup. The MWI and MCW values for segments with FFR ≤ 0.75 were significantly lower compared to those with FFR 0.76–0.80, and FFR > 0.80. *p < 0.05, **p < 0.01. FFR, fractional flow reserve; MCW, myocardial constructive work; MWI, myocardial work index.

For both the total-vessel and single-vessel stenosis groups, the MWI and MCW in patients with FFR ≤ 0.75 were significantly lower than those in patients with FFR > 0.75 (Table 2). There were no significant differences regarding MWI or MCW between vessels with FFR ≤ 0.80 and those with FFR > 0.80 in both the total-vessel and single-vessel stenosis groups (Supplementary Table 1). Figure 2 illustrates MWI and MCW between LV segments perfused by vessels with FFR ≤ 0.75, 0.76–0.80, and >0.80 in total-vessel and single-vessel stenotic patients, respectively. The MWI and MCW in LV segments perfused by vessels with FFR ≤ 0.75 tended to be smaller than those with FFR 0.76–0.80, and >0.80 significantly (*p* < 0.05).

**Table 2:** Comparing MW parameters and GLS between the FFR ≤ 0.75 and FFR > 0.75 groups.

**Characteristics**	**Total**	**FFR ≤0.75**	**FFR > 0.75**	***P-*value**
**Total vessels**				
Number of vessels	92	18	74	
GLS, %	−17.3 ± 2.4	−15.8 ± 2.2	−17.7 ± 2.3	0.002
MWI, mmHg%	1,782.2 ± 358.2	1,522.3 ± 277.0	1,845.4 ± 348.4	0.000
MWE, %	92.8 ± 4.5	92.1 ± 4.0	92.9 ± 4.6	0.517
MCW, mmHg%	2,127.9 ± 389.7	1,851.2 ± 262.4	2,195.2 ± 387.1	0.001
MWW, mmHg%	140.9 ± 98.1	150.6 ± 99.1	138.5 ± 98.4	0.641
**Single vessel involved**				
Number of vessels	57	11	46	
GLS, %	−17.4 ± 2.3	−16.0 ± 2.3	−17.8 ± 2.2	0.024
MWI, mmHg%	1,796.4 ± 368.3	1,463.8 ± 323.0	1,876.0 ± 334.8	0.001
MWE, %	93.1 ± 4.6	92.3 ± 4.5	93.3 ± 4.7	0.525
MCW, mmHg%	2,137.0 ± 398.6	1,794.9 ± 308.4	2,218.8 ± 375.7	0.001
MWW, mmHg%	136.3 ± 106.0	155.5 ± 117.5	131.8 ± 103.9	0.510

*Data are expressed as mean ± SD when appropriate. FFR, fractional flow reserve; GLS, global longitudinal strain; MCW, myocardial constructive work; MW, myocardial work; MWE, myocardial work efficiency; MWI, myocardial work index; MWW, myocardial wasted work*.

Intra- and inter-observer variabilities were measured in 15 randomly selected patients. The intra-class correlation coefficient (ICC) for intra-observer variability was 0.95 (95% CI: 0.86–0.98) for MWI and 0.90 (95% CI: 0.73–0.97) for MCW. The inter-observer ICC was 0.83 (95% CI: 0.56–0.94) for MWI and 0.89 (95% CI: 0.71–0.96) for MCW.

### Correlations Between FFR and MW

**Figure 3:**
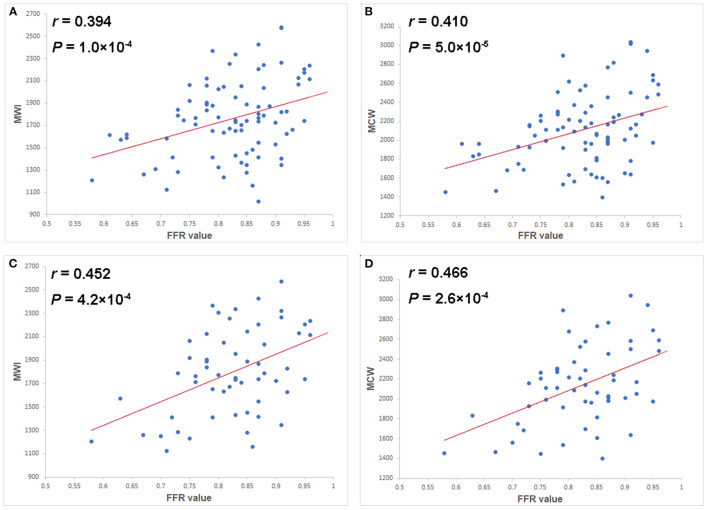
Scatter plots of the MWI **(A)** and MCW **(B)** in the total-vessel stenosis group, and of the MWI **(C)** and MCW **(D)** in the single-vessel stenosis subgroup vs. FFR values. Both MWI and MCW showed mild but significant positive correlations with FFR values (*r* = 0.394*, p* = 0.000 and *r* = 0.410, *p* = 0.000 in total group; *r* = 0.452, *p* = 0.000 and *r* = 0.466, *p* = 0.000 in single vessel group). FFR, fractional flow reserve; MCW, myocardial constructive work; MWI, myocardial work index.

As shown in Figure 3, both MWI and MCW showed a significant positive correlation with FFR values in the total-vessel (*r* = 0.394, *p* = 0.000; *r* = 0.410, *p* = 0.000) and the single-vessel stenosis groups (*r* = 0.452, *p* = 0.000; *r* = 0.466, *p* = 0.000). Other MW indices were not significantly correlated with FFR values. GLS showed a significant negative correlation with FFR values in both total-vessel group (*r* = −0.306, *p* = 0.003) and single-vessel stenosis group (*r* = −0.273, *p* = 0.04).

### Diagnostic Values of Strain Related Variables for Diagnosing Segments With low FFR

**Figure 4:**
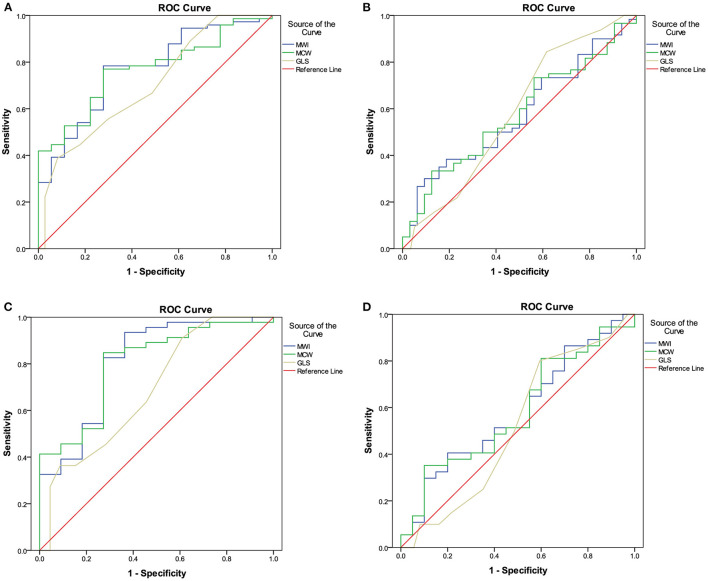
ROC analyses of MW parameters for the diagnosis of left ventricular segments with FFR values ≤ 0.75 **(A)** and ≤ 0.80 **(B)** in the total-vessel group, and FFR values ≤ 0.75 **(C)** and ≤ 0.80 **(D)** in the single-vessel subgroup. **(A)** The best MWI cutoff value to detect LV segments perfused by vessels with an FFR value ≤ 0.75 in the total-vessel group was 1,623.7 mmHg% [sensitivity, 78.4%; specificity, 72.2%; AUC, 0.768 (0.653–0.883)]. **(B)** In the total-vessel group, the best cutoff value was 1,962.4 mmHg% [77.0%; 72.2%; 0.767 (0.661–0.872)] for MCW. **(C)** In the single-vessel subgroup, the best cutoff value was 1,412.1 mmHg% [93.5%; 63.6%; 0.808 (0.652–0.965)] for MWI. **(D)** In the single-vessel subgroup, the best cutoff value was 1,943.3 mmHg% [(84.8%; 72.7%; 0.800 (0.657–0.943)] for MCW. FFR, fractional flow reserve; MW, myocardial work; ROC, receiver operator characteristics.

ROC curve analysis was used to determine the sensitivity and specificity of the MW parameters to detect segments perfused by vessels with an FFR ≤ 0.75 and an FFR ≤ 0.80 (Figure 4). ROC curve analysis showed that MWI and MCW had a good diagnostic performance for the prediction of FFR ≤ 0.75 in both total-vessel group and single-vessel stenosis group [area under the curve (AUC) between 0.77 and 0.81, *p* < 0.01]. The optimal cutoff values of MWI and MCW were 1,623.7 mmHg% (sensitivity 78.4%, specificity 72.2%) and 1,962.4 mmHg% (sensitivity 77.0%, specificity 72.2%) in the total-vessel group and 1,412.1 mmHg% (sensitivity 93.5%, specificity 63.6%) and 1,943.3 mmHg% (sensitivity 84.8%, specificity 72.7%) in the single-vessel group (Table 3).

**Table 3:** Diagnostic values of strain-related variables for detecting significant myocardial ischemia using FFR as the gold standard.

	**Cutoff**	**AUC (95% CI)**	**Sensitivity**	**Specificity**	**Accuracy**	***P-*value**
**Total group**						
Cut-off of FFR 0.75						
GLS, (%)	−14.5	0.707 (0.574–0.841)	0.389	0.919	0.654	0.007
MWI, (mmHg%)	1,623.7	0.768 (0.653–0.883)	0.784	0.722	0.753	0.000
MWE, (%)	92.4	0.581 (0.435–0.725)	0.676	0.556	0.616	0.286
MCW, (mmHg%)	1,962.4	0.767 (0.661–0.872)	0.770	0.722	0.746	0.000
MWW, (mmHg%)	136.4	0.541 (0.385–0.696)	0.556	0.649	0.603	0.595
Cut-off of FFR 0.80						
GLS, (%)	−18.5	0.584 (0.466–0.702)	0.844	0.383	0.614	0.184
MWI, (mmHg%)	2,065.4	0.576 (0.456–0.695)	0.300	0.906	0.603	0.235
MWE, (%)	95.6	0.557 (0.440–0.675)	0.400	0.781	0.591	0.367
MCW, (mmHg%)	2,350.3	0.577 (0.457–0.696)	0.333	0.875	0.604	0.228
MWW, (mmHg%)	83.6	0.503 (0.383–0.623)	0.750	0.367	0.559	0.961
**Single-vessel stenosis group**						
Cut-off of FFR 0.75						
GLS, (%)	−18.5	0.696 (0.532–0.860)	0.909	0.391	0.650	0.045
MWI, (mmHg%)	1,412.1	0.808 (0.652–0.965)	0.935	0.636	0.786	0.002
MWE, (%)	91.7	0.587 (0.398–0.776)	0.783	0.455	0.619	0.374
MCW, (mmHg%)	1943.3	0.800 (0.657–0.943)	0.848	0.727	0.788	0.002
MWW, (mmHg%)	152.9	0.551 (0.345–0.757)	0.455	0.783	0.619	0.599
Cut-off of FFR 0.80						
GLS, (%)	−18.5	0.521 (0.367–0.675)	0.800	0.405	0.603	0.796
MWI, (mmHg%)	1,935.7	0.581 (0.426–0.736)	0.405	0.800	0.603	0.316
MWE, (%)	96.3	0.561 (0.412–0.711)	0.324	0.900	0.612	0.447
MCW, (mmHg%)	2335.8	0.581 (0.426–0.736)	0.351	0.900	0.626	0.316
MWW, (mmHg%)	42.6	0.516 (0.363–0.669)	1.000	0.135	0.568	0.841

*AUC, area under the curve; CI, confidence interval; GLS, global longitudinal strain; FFR, fractional flow reserve; MCW, myocardial constructive work; MWE, myocardial work efficiency; MWI, myocardial work index; MWW, myocardial wasted work; ROC, receiver operator characteristics*.

### MW Parameters Before and After Percutaneous Coronary Intervention

There were 18 vessels with FFR ≤ 0.75. In those patients who received PCI, echocardiography was repeated after PCI in 13 of the 18 vessels (72.2%). In total, 72.2% of the patients had both preoperative and postoperative images. MWI (1,514.6 ± 294.6 vs. 1,809.8 ± 397.5 mm Hg%*, p* = 0.010), MCW (1,863.0 ± 265.8 vs, 2,187.6 ± 461.6 mmHg%, *p* = 0.016) and GLS (-15.7 ± 2.2 vs, −17.3 ± 2.7 mmHg%*, p* = 0.029) increased significantly at a median of 3 days (range 1–7 days) after intervention. There were no significant differences in MWE and MWW when comparing before and after PCI (*p* > 0.05).

## Discussion

In the current work, we found that the MWI and MCW were significantly lower in LV segments perfused by vessels with an FFR ≤ 0.75 than in those with an FFR > 0.75. MWI and MCW were of good diagnostic value for identifying LV segments of low FFR, especially in patients with single-vessel stenosis.

To our knowledge, this is the first study to report the diagnostic accuracy and potential advantages of MW in diagnosing ischemic myocardial segments confirmed by FFR. Coronary angiography, mostly defining CAD as having ≥70% narrowing in one or more coronary arteries, has been used as the standard approach to validate MW (8, 12, 30). However, the revascularization procedures are based not only on the coronary anatomy but also on the functional status of the lesion. In this regard, FFR has become a reliable measure (6, 16). Therefore, in the current study FFR was used as the gold standard for the assessment of the functional severity of coronary artery stenosis. We found that reduced MW indices were associated with FFR ≤ 0.75. Interestingly, using the two different diagnostic criteria (70% stenosis vs. FFR ≤ 0.75), the AUCs of ROC curves for MWI and MCW were similar (12, 30). However, the optimal cutoffs of MWI for detecting FFR ≤ 0.75 in both the total-vessel group and single-vessel stenosis group seem to be lower than the cutoff for detecting ≥ 70% stenosis (1,623.7 mmHg % and 1,412.1 mmHg % from the current study vs. around 1,800 mmHg% in literature) (12, 30). This disparity reflects a difference between functional significance and the anatomical degree of coronary artery stenosis.

In previous studies, compared to LVEF and GLS, MW has exhibited a better sensitivity and accuracy in detecting patients with single- or multivessel CAD (31). Among all MW indices, the MWI and MCW are the more significant predictors for detecting ischemia (8, 30). Our results also support MWI and MCW as having a better diagnostic value than LV strain. Speckle tracking by echocardiography can also be used to diagnose CAD (32). Previous study has found that GLS was impaired in all CAD patients, but only was significantly impaired in severe CAD patients with left main or three-vessel CAD (33). In our study, most patients suffered from single-vessel lesions, and in general, the sample size was small. These factors may account for the weak statistical differences in GLS between groups. Interestingly, our study found that the diagnostic specificity of GLS was high in the total-vessel stenosis group but low in the single-vessel stenosis group in detecting left ventricular segments with an FFR ≤ 0.75. This may be because more segmental LV strains were impacted in the total-vessel stenosis group. However, in single-vessel stenosis group LV global strain has a low specificity (39.1%) (32). In contrast, MWI and MCW showed relatively good specificities (63.6–72.7%) in single-vessel stenosis group for identifying LV segments perfused by vessels with an FFR ≤ 0.75. This may partly be due to MW being used to assess the myocardial contraction in the context of afterload, which makes it less load-dependent (34).

We also found in current study that the AUCs of MWI and MCW for detecting FFR ≤ 0.75 in the single-vessel subgroup were higher than those in the total-vessel group. Similarly, Ishigaki et al. found that the incremental diagnostic value of speckle-tracking parameters for detecting LAD stenosis in a single-vessel group was more significant than in a multi-vessel group (35). Impaired MW parameters suggest early subclinical myocardium dysfunction (31, 36, 37). Dysfunction in the myocardium that was perfused by other vessels may affect the calculation of MWI and MCW in the single-vessel territory. For example, the presence of RCA and/or LCX lesions in patients with LAD disease may affect the value of regional MWI and MCW corresponding to LAD. Furthermore, the analysis of regional MW may allow better screening of single-vessel stenosis.

Both FFR cutoff values of 0.75 and 0.80 have been used in previous invasive diagnostic tests (38). Cases of FFR 0.76–0.80 may fall into a gray-zone which includes lesions that are not truly ischemic (39). Our results showed a significant difference if FFR ≤ 0.75 rather than ≤ 0.80, which is consistent with the finding from Nishi T et al. (5).

In our cohort, the MWI and MCW increased significantly after intervention. Recent studies have demonstrated in patients with anterior ST-elevation myocardial infarction who received PCI that MW is an independent predictor of LV recovery and early adverse LV remodeling (40, 41). MW may be a useful tool to monitor the myocardial function during the short- and long-term follow-up of patients after PCI.

### Limitations

This study has several limitations that should be mentioned. Patients were recruited from a single tertiary center and may not reflect the general population (5, 6, 17). The ≤ 0.75 FFR subgroup comprised only 11 patients in the single vessel group. The cut-off value of MWI in single vessel group needs to be proved in a larger sample size study. A larger number of patients is also needed to investigate the diagnostic value of MWs for segments with RCA and LCX stenosis. From the current result, the superiority of MWI and MCW over GLS in the single-vessel group suggested a role of early systolic lengthening and post-systolic shortening. Peak strain is determined by afterload concurrently with contraction, while segmental systolic function in early mid-systole is impaired reflecting the impact exerted by a higher afterload in this phase. However, post-systolic index was not included in our study design, therefore we cannot address the diagnostic value of this index in current study. It is certainly an interesting direction for our future research.

## Conclusion

Regional MW assessed by echocardiography are promising non-invasive parameters for the quantification of the functional severity of intermediate coronary stenosis, especially in patients with single-vessel stenosis. MW parameters at rest exhibited good diagnostic value in detecting significant myocardial ischemia.

## Data Availability Statement

The raw data supporting the conclusions of this article will be made available by the authors, without undue reservation.

## Ethics Statement

The studies involving human participants were reviewed and approved by Ethics Committee of Beijing Hospital (Reference Number: 2020BJYYEC-021-02). Our study is a post-hoc analysis of data from an IRB-approved prospective clinical trial (NCT03905200). The patients/participants provided their written informed consent to participate in this study.

## Author Contributions

YG, CY, and FW are the major contributors in writing the manuscript. YG and HZ analyzed the echocardiograms. XW, ZZ, and XM collected the patient information. ZP, XL, and SN revised the manuscript carefully. FW conceived the study and supervised the project. All authors read and approved the final manuscript.

## Funding

This work was supported by grants from the National Key R&D Program of China (2020YFC2008100/2020YFC2008106) and the Beijing Hospital Research Project (No. BJ-2019-133).

## Conflict of Interest

The authors declare that the research was conducted in the absence of any commercial or financial relationships that could be construed as a potential conflict of interest.

## Publisher's Note

All claims expressed in this article are solely those of the authors and do not necessarily represent those of their affiliated organizations, or those of the publisher, the editors and the reviewers. Any product that may be evaluated in this article, or claim that may be made by its manufacturer, is not guaranteed or endorsed by the publisher.
