# Corrigendum: Regional myocardial work measured by echocardiography for the detection of myocardial ischemic segments: a comparative study with invasive fractional flow reserve

**DOI:** 10.3389/fcvm.2024.1383226

**Published:** 2024-03-11

**Authors:** Ying Guo, Chenguang Yang, Xiang Wang, Zuowei Pei, Huolan Zhu, Xuyang Meng, Ziyu Zhou, Xiaotong Lang, Sun Ning, Ruisheng Zhang, Fang Wang

**Affiliations:** ^1^ Department of Cardiology, Beijing Hospital, National Center of Gerontology, Beijing, China; ^2^ Institute of Geriatric Medicine, Chinese Academy of Medical Sciences, Beijing, China; ^3^ Graduate School of Peking Union Medical College, Chinese Academy of Medical Sciences, Beijing, China; ^4^ Department of Gerontology, Shanxi Provincial People’s Hospital, Shanxi Provincial Clinical Research Center for Geriatric Medicine, Xi’An, China

**Keywords:** myocardial work, fractional flow reserve, regional myocardial work, coronary artery disease, single-vessel stenosis

A Corrigendum on Regional myocardial work measured by echocardiography for the detection of myocardial ischemic segments: a comparative study with invasive fractional flow reserve By Guo Y, Yang C, Wang X, Pei Z, Zhu H, Meng X, Zhou Z, Lang X, Ning S, Zhang R, Wang F (2022). Front Cardiovasc Med. 9:813710. doi: 10.3389/fcvm.2022.813710

In the published article, there was an error regarding the affiliation for Chenguang Yang and Fang Wang. As well as having affiliations 1 and 2, they should also have “Graduate School of Peking Union Medical College, Chinese Academy of Medical Sciences, Beijing, China”.

The authors apologize for this error and state that this does not change the scientific conclusions of the article in any way. The original article has been updated.

