# Supplementary material for: Regional Myocardial Work Measured by Echocardiography for the Detection of Myocardial Ischemic Segments: A Comparative Study With Invasive Fractional Flow Reserve
Source: Front Cardiovasc Med. 2022 Mar 16;9:813710. doi: 10.3389/fcvm.2022.813710 (PMC8965858; doi:10.3389/fcvm.2022.813710)
Supplement: Supplementary file 1 [file Table_1.DOCX]

Supplementary Material

**Supplementary Table. Comparing MW parameters between FFR ≤ 0.80 and FFR > 0.80 groups**

| **Characteristics** | **Total** | **FFR** ≤ **0.80** | **FFR** > **0.80** | ***P* value** |
| --- | --- | --- | --- | --- |
| Total vessels |  |  |  |  |
| Number of vessels | 92 | 32 | 60 |  |
| GLS, % | -17.3±2.4 | -16.8±2.1 | -17.6±2.5 | 0.181 |
| MWI, mmHg% | 1782.2±358.2 | 1715.0±315.4 | 1818.0±376.7 | 0.191 |
| MWE, % | 92.8±4.5 | 92.7±3.7 | 92.8±4.9 | 0.922 |
| MCW, mmHg% | 2127.9±389.7 | 2058.3±344.3 | 2165.0±409.8 | 0.213 |
| MWW, mmHg% | 140.9±98.1 | 133.7±75.8 | 144.7±108.5 | 0.613 |
| Single vessel involved |  |  |  |  |
| Number of vessels | 57 | 20 | 37 |  |
| GLS, % | -17.4±2.3 | -17.4±2.1 | -17.5±2.5 | 0.868 |
| MWI, mmHg% | 1796.4±368.3 | 1718.1±355.7 | 1838.7±372.7 | 0.242 |
| MWE, % | 93.1±4.6 | 93.0±3.9 | 93.2±5.0 | 0.864 |
| MCW, mmHg% | 2137.0±398.6 | 2052.5±374.4 | 2182.6±408.8 | 0.243 |
| MWW, mmHg% | 136.3±106.0 | 129.3±79.3 | 140.1±118.8 | 0.715 |

Data are expressed as mean ± SD when appropriate. FFR fractional flow reserve, GLS global longitudinal strain, MCW myocardial constructive work, MW myocardial work, MWE myocardial work efficiency, MWI myocardial work index, MWW myocardial wasted work
